# Stage-dependent prognostic shift in mismatch repair-deficient tumors: Assessing patient outcomes in stage II and III colon cancer

**DOI:** 10.3389/fonc.2022.853545

**Published:** 2022-08-30

**Authors:** Kjersti Elvestad Hestetun, Nina Benedikte Rosenlund, Luka Stanisavljević, Olav Dahl, Mette Pernille Myklebust

**Affiliations:** ^1^ Department of Clinical Science, University of Bergen, Bergen, Norway; ^2^ Department of Oncology, Haukeland University Hospital, Bergen, Norway

**Keywords:** microsatellite instability, mismatch repair (MMR), colon cancer, prognosis, stage

## Abstract

**Introduction:**

Deficient mismatch repair (dMMR) or high microsatellite instability (MSI-H) is associated with an improved prognosis in colon cancer stage II but poor prognosis in stage IV colon cancer. The clinical significance of dMMR in colon cancer stage III is not established.

**Methods:**

Tissue microarrays (TMAs) from 544 patients with colon cancer stage II and III with clinicopathological and survival data were stained for mismatch repair (MMR) proteins, CD3, CD8, and programmed death ligand-1 (PD-L1), and programmed death ligand- 1 (PD-L1). Patient outcomes were reviewed.

**Results:**

In stage III colon cancer, dMMR was a marker of poor disease-free survival (DFS) (Kaplan–Meier, mean survival in months: dMMR: 28.76 (95% CI 18.46–39.05) vs. pMMR 40.91 (37.20–44.63), p=0.014, multivariate Cox regression: hazard ratio (HR) 4.17 (95% CI 2.02–8.61), p<0.001). In stage II colon cancer, there was a tendency toward improved DFS for dMMR patients (dMMR: 57.14 (95% CI 54.66–59.62) vs. pMMR 53.54 (95% CI 51.48–55.60), p=0.015, multivariate Cox regression HR 0.24 (95% CI 0.06-1.04), p=0.057). CD3, CD8, and PD-L1 expression was not associated with prognosis of dMMR patients. Multivariate Cox regression analysis showed a significant interaction between the MMR phenotype and stage (p=0.001).

**Conclusion:**

dMMR is associated with an improved prognosis in stage II colon cancer but is no longer associated with a favorable prognosis in stage III colon cancer.

## 1 Introduction

Deficient mismatch repair (dMMR) or high microsatellite instability (MSI-H) is a marker of improved prognosis in stage II colon cancer (1). This phenomenon has been attributed to the beneficial immune response associated with dMMR tumors (2). However, in stage IV colorectal cancer (CRC), several studies demonstrate that a dMMR phenotype is a marker of poor prognosis (1, 3, 4). In stage III, MMR phenotyping is currently only used to detect Lynch syndrome and the effect of dMMR on prognosis is controversial (5).

The reason for the contrasting prognostic impact of the MMR phenotype in different stages of colon cancer is not established. A dMMR phenotype gives rise to a high mutational burden, particularly insertion and deletions (indels), which again leads to an increased expression of neoantigens and higher density of tumor-infiltrating lymphocytes (TILs) (6, 7). A high infiltration of CD3+ (pan T- cell marker) and CD8+ (cytotoxic T-cell marker) cells is viewed as a manifestation of a favorable immune response and a beneficial prognostic marker in colon cancer (8).

Tumors might evade their immunosuppressive environment by exploiting existing immune checkpoints that in normal physiology are used to maintain self-tolerance. Programmed death–ligand 1 (PD-L1) is a transmembrane protein typically expressed on hemopoietic and antigen-expressing cells. During immune regulation, PD-L1 binds to programmed death-1 receptor (PD-1) on cytotoxic T cells and other immune cells. It has been suggested that increased PD-L1 expression in tumor cells represents an escape from immune surveillance, allowing for the spread of tumor cells and decreased cancer-specific survival in dMMR tumors (9).

Few studies have described the prognosis of dMMR in stage III colon cancer. Improving the prognostication of dMMR colon cancer is highly warranted to identify patients at risk of colon cancer relapse that might benefit from immunotherapy. The main goal of this study was to analyze the prognosis of dMMR stage III colon cancer and study the prognostic interplay between MMR status, tumor cell PD-L1 expression, and density of CD3+ and CD8+ lymphocytes.

## 2 Methods

### 2.1 Patient cohorts

The study cohorts have been described previously (10). It includes tissue from the primary tumors of all included patients, 544 in total, with colon cancer stage II and III from two clinical studies. The 276 patients from the Norwegian Gastrointestinal Cancer Group (NGICG) material were included between 1993 and 1996 (11). They were randomized to receive adjuvant chemotherapy with fluorouracil/levamisole after surgery or to surgery only. The remaining 268 patients were included from the The Haraldsplass Deaconess Hospital (HDH)–material; a population-based cohort with colon cancer patients recruited from 2007 to 2011 (12, 13). The patients from the NGICG and HDH series have similar clinicopathological characteristics, as shown in **Table 1**. None of the patients received immunotherapy or radiotherapy. The molecular biomarkers analyzed in this study were not used for treatment selection. Median follow-up time was 5 years for both the HDH cohort and the NGICG cohort.

**Table:** Table 1 Patient characteristics (%).

	Number of patients (%)
**Sex**	
Male	274 (50.4%)
Female	270 (49.6%)
**MMR phenotype^a^**	
dMMR	105 (19.3%)
pMMR	377 (69.3%)
**PD-L1^b^**	
Negative (<1%)	361 (66.4%)
Positive 1-49%	68 (12.5%)
Positive >50%	6 (1.1%)
**Intraepithelial CD3/CD8** **T-cell density score^c^**	
0 (no positive cells)	78 (14.3%)/116 (21.3%)
1 (low)	138 (25.4%)/112 (20.6%)
2 (high)	129 (23.7%)/117 (21.5%)
3 (very high)	125 (23.0%)/107 (19.7%)
**Stromal CD3/CD8 T-cell density score (mean/SD)**	24.5 (16.4)/7.3 (8.0)
**Tumor grade^d^**	
Low tumor grade	430 (79.0%)
High tumor grade	107 (19.7%)
**Stage**	
UJCC stage II	338 (62.1%)
UJCC stage III	206 (37.9%)
**Chemotherapy^e^**	
Adjuvant chemo.	189 (34.7%)
No chemotherapy	354 (65.1%)
**Mean age (range)**	67.5 (28.0-93.0)

a: Mismatch repair (MMR): Data missing from 62 patients (11.4%). b: Programmed cell death ligand-1 (PD-L1): Results missing from 109 patients (20.0%). c: Tumor-infiltrating lymphocytes (TILs): Results missing from 74 (14%)/92 (17%) patients. d: Grade: Data missing from 7 patients (1.3%). e: Chemotherapy: Data missing from 1 patient.

### 2.2 Tissue microarrays and immunohistochemistry

Immunohistochemistry (IHC) was performed on tissue microarrays (TMAs) containing tissue cores from primary tumors as well as adjacent normal colon mucosa when available. Cores, three from each case, were 1.0 mm in diameter and obtained from formalin-fixed and paraffin-embedded tissue. For all protocols, 2–5-µm tissue sections were sectioned from TMA blocks, deparaffinated, and rehydrated. After IHC staining, the slides were counterstained with hematoxylin, dehydrated, and mounted.

The CD8 IHC was performed on a Ventana BenchMark Ultra platform with target retrieval buffer CC1 (36 min) using the anti-CD8 mouse monoclonal antibody (Ab) clone C8/144B (Dako, P/N M7103) at 1:100 dilution with 32-min incubation time. The Ventana UltraView DAB Detection Kit (P/N 760-500, Roche Diagnostics, Indiana, USA) and Amplification Kit (P/N 760-080, Roche Diagnostics), were used. CD3 IHC was performed on a Ventana Discovery instrument with target retrieval buffer CC1, the extended HIER protocol and the anti-CD3 rabbit monoclonal Antibody from Roche Diagnostics GmbH (2GV6), 790-4341 (ready to use) with 60-min incubation time. The detection kit was Ventana Discovery UltraMap anti-Rb HRP (RUO, P/N 760-4315, Roche Diagnostics) in conjunction with Ventana Discovery ChromoMap DAB Kit (RUO, P/N 760-159, Roche Diagnostics). PD-L1 IHC was performed on a BenchMark Ultra platform with target retrieval using CC1 buffer for 64 min and the Ventana anti-PD-L1 rabbit monoclonal Ab, clone SP 263 (P/N 741-4905, Roche Diagnostics) with 16-min incubation time and Ventana OptiView DAB IHC Detection Kit (P/N 760-700, Roche Diagnostics). MMR and CDX2 IHC has been described previously (10).

### 2.3 Scoring of tissue microarrays

#### 2.3.1 CD3 and CD8

The densities of CD3+ and CD8+ T lymphocytes were both assessed within the tumor margins (intraepithelial TILs) and stroma <100 μm from the tumor margin (stromal lymphocytes). In cases of intratumoral heterogeneity, the cylinder with the highest density of lymphocytes was included in the analysis. Two of the authors (MPM and KEH) scored the TMAs. The scores were semiquantitative and based on the density of positive T cells. We divided the intraepithelial lymphocytes into four groups based on the density of positive lymphocytes: 0 = no positive cells, 1= low, 2 = high, and 3 = very high density. For the stromal lymphocytes, we observed that most cases had a low density of lymphocytes, and few cases had a very high density. The score was therefore made from 0 to 100 to detect variations in the lower segment. For presentation in Kaplan–Meier plots, the stromal scores were divided into four groups based on score quartiles. A total TIL density score was computed for multivariate analyses. For the stromal scores and the intraepithelial scores to contribute equally to the combined score, the stromal scores were divided into four groups based on quartile scores and the given score 0–3. The sum of the intraepithelial CD3+ TILs (0–3), intraepithelial CD8+ TILs (0-3), stromal CD3+ TILs (0–3) and stromal CD8+ TILs (0–3) were used as the total TIL score (TIL density score, 0–12).

#### 2.3.2 Programmed death ligand-1

PD-L1 expression was assessed by authors KEH and MPM as recommended by the Agilent Dako Interpretation Manual (originally made for NSCLCs). This scoring method is compatible with the tumor proportion score (14). PD-L1 expression was divided into three groups based on the percentage of PD-L1- positive tumor cells: <1%: no PD-L1 expression. 1%–49%: positive PD-L1 expression, ≥50%: highly positive PD-L1 expression. Positive staining was defined as partial or complete cell membrane staining of any intensity that was perceived distinct from cytoplasmic staining. Cytoplasmic staining was excluded from the scoring. Only viable tumor cells were scored. Immune cells, normal cells, and necrotic cells were excluded from the scoring. Staining in tumor-associated immune cells was recorded separately.

#### 2.3.3 CDX2

Cases were regarded as CDX2 positive if >50% of tumor cells exhibited nuclear CDX2 staining (10).

#### 2.3.4 Mismatch repair protein expression scoring

Negative MMR protein staining was defined as <5% positive tumor cells in the presence of positive staining in internal positive control cells in the same tissue core (normal colon epithelium or stromal cells). The MMR protein staining was nuclear. Cores with negative staining for both tumor cells and internal control cells generated a missing staining result. MMR-deficient cases (dMMR) were defined as cases with negative staining for MLH1 + PMS2, MSH2 + MSH6, MSH6 alone, or PMS2 alone. Cases with positive MMR protein staining were defined as MMR proficient (pMMR). We have validated the MMR protein staining in whole tissue sections (10).

### 2.4 Statistics

Disease-free survival (DFS) was defined as time from surgery until recurrence. Overall survival (OS) was defined as time from surgery until death of any cause. Two-sided p-values are reported and values <0.05 were considered statistically significant. P-values were not adjusted for multiple testing. Cox regression models were fitted using the enter method with the clinically relevant factors included in the analyses. The MMR phenotype-by-stage interaction was tested by adding a cross-product term of indicator variables for the MMR phenotype and stage to the Cox regression model. The assumption of proportional hazards was assessed by the inspection of log–log HR (hazard ratio) and HR of Cox regression models stratified on the categorical variables and Schoenfeld residuals (continuous variables). Differences in means was tested using the T test (for normally distributed variables) or Mann–Whitney U-test. Median follow-up time was calculated by the reverse Kaplan–Meier method. Statistical analyses were performed using IBM SPSS Statistics for Windows (v25.0)

### 2.5 Ethics

Study protocols were approved by The Regional Committee for Medical Research Ethics of Western Norway and the Data Inspectorate for National Registries (REK 1992-55.92, REK 15666). All patients signed informed consent.

## 3 Results

### 3.1 Patient characteristics

**Figure 1:**
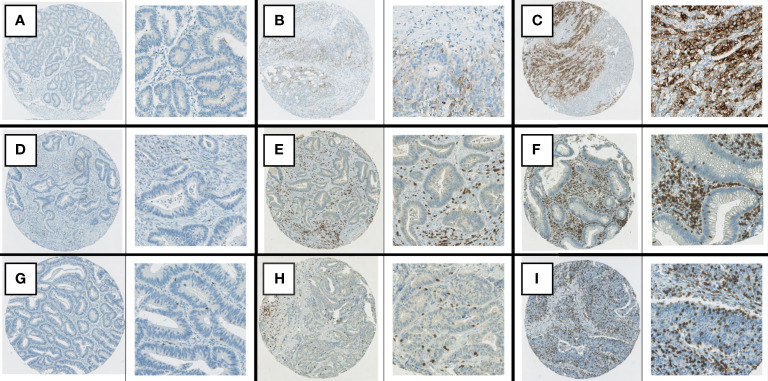
Examples of immunohistochemical staining. **(A)** PD-L1, negative staining in tumor cells. **(B)** PD-L1, positive staining in >1% but <50% of tumor cells. **(C)** PD-L1, positive staining in > 50% of tumor cells. **(D)** CD8 staining: stromal score in lowest quartile, TIL score 0. **(E)** CD3 staining, stromal score in second highest quartile, TIL score 1+. **(F)** CD3 staining, stromal score in highest quartile, TIL score 2+. **(G)** CD8 staining, TIL score 1+. **(H)** CD3 staining, TIL score 2+. **(I)** CD8 staining, TIL score 3 +.

In total, 105 patients (19.3%) had dMMR tumors and 377 patients (69.3%) had pMMR tumors (**Table 1**). Insufficient staining results was seen in 62 patients (11.4%). There was a higher frequency of dMMR in stage II versus stage III (24.6% vs. 10.7%). PD-L1 positive staining in 1-49% of tumor cells was observed in 68 patients (12.5%) (**Figure 1**). Only six patients (1.1%) had ≥50% PD-L1-positive tumor cells; therefore, all patients with positive PD-L1 staining in ≥1% of tumor cells were defined as PD-L1 positive for further analyses. The densities of CD3+ and CD8+ intraepithelial and stromal TILs are described in **Table 1**.

Missing staining results were due to technical issues: insufficient staining in cells used as internal positive controls (MMR proteins), necrosis, tissue core detached from slide during staining, or few tumor cells available for scoring. Goodness-of-fit analyses have been made for all markers [**Supplementary** and (10)] and showed no statistically significant differences in the distribution of clinicopathological variables between the original study cohort and patients with valid staining results.

The number of examined lymph nodes and number of positive lymph nodes for stage III dMMR and pMMR tumors were assessed from pathology reports and were found not to be different in dMMR patients versus pMMR. For stage III patients in the HDH cohort, the mean number of examined lymph nodes was 15.92 (SD 3.4) for patients with dMMR versus 16.89 (SD 5.4) for patients with pMMR (p=0.53). The mean number of positive lymph nodes for stage III was 2.38 (SD 1.9) for dMMR tumors and 3.14 (SD 2.3) for patients with pMMR (p=0.23). The mean number of examined lymph nodes for stage III colon cancer in the NGICG cohort was 8.00 (SD 4.6) for patients with dMMR versus 9.73 (SD 6.8) for patients with pMMR (p=0.68),. The mean number of positive lymph nodes for stage III was 3.67 (SD 3.2) for dMMR tumors and 3.30 (SD 4.1) for patients with pMMR (p=0.48).

### 3.2 Mismatch repair proteins and prognosis

**Figure 2:**
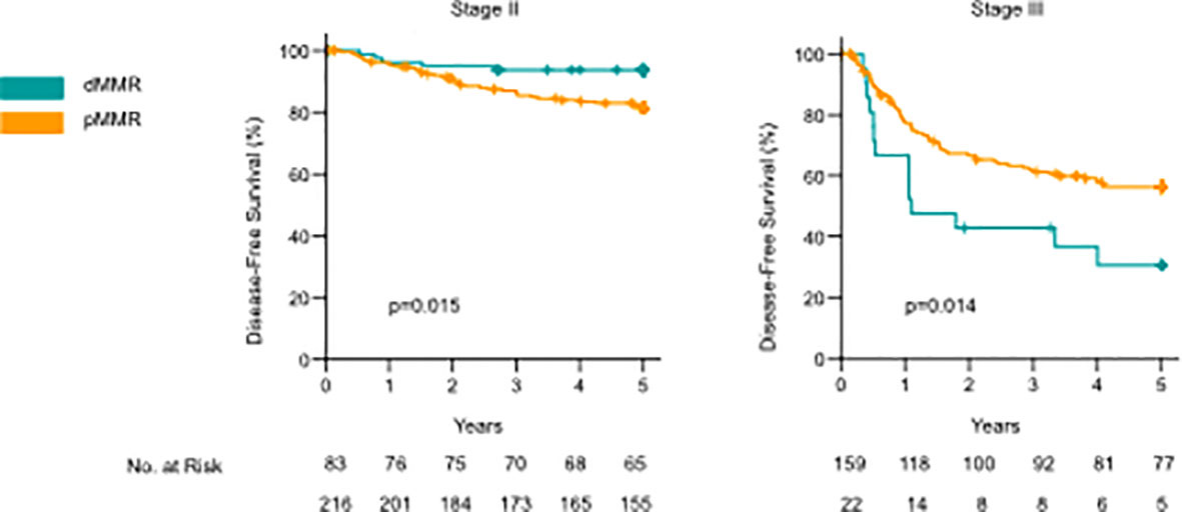
Kaplan–Meier curves comparing disease-free survival (DFS) and overall survival between dMMR and pMMR cases for stage II and stage III colon cancer. P-values calculated by the log-rank test.

MMR deficiency was significantly associated with lower stage, CDX2 negativity, right-sided cancer, mucinous- or signet-ring histology, PD-L1 positivity, and a high density of CD3+ and CD8+ TILs, both stromal and intraepithelial (**Table 2**). When analyzing stage II and III together, there was no statistically significant difference in the mean DFS time between dMMR and pMMR patients (51.23 (95% CI 47.54-54.93) vs. 48.22 (95% CI 46.15-50.29) months, p=0.074) or mean OS (49.82 (95% CI 46.23-53.42) vs. 50.29 (95% CI 48.5-52.1), p=0.957). dMMR was a marker of improved mean DFS in stage II colon cancer when compared to pMMR (57.14 (95% CI 54.66-59.62) vs. 53.54 (95% CI 51.48-55.61 months), p=0.015), but no difference was observed for mean OS (53.95 (95%CI 50.71-57.19) vs. 54.12 (52.24-56.02), p=0.890) (**Figure 2**). In contrast, in stage III patients, the dMMR phenotype was a marker of poor mean DFS and OS compared to patients with pMMR [DFS: 28.76 (95% CI 18.46-39.05) vs. 40.91 (37.20-44.63), p=0.014], (OS: 34.27 (95% CI 24.74-43.80) vs. 45.07 (95% CI 41.94-48.21), p=0.018) (**Figure 2**). These data show that dMMR is a marker of poor prognosis in stage III colon cancer but a marker of improved prognosis in stage II.

**Table 2:** Associations between MMR phenotype, PD-L1 expression, and other markers.

VARIABLE	PD-L1 expression^a^ (n = 435)			MMR phenotype (n = 482)		
	Positive, n (%)	Negative, n (%)	p-value^b^	dMMR n (%)	pMMR n (%)	p-value^b^
**Sex**						
Female	33 (16.0%)	173 (84.0%)	0.612	58 (24.9%)	175 (75.1%)	0.123
Male	41 (17.9%)	188 (82.1%)		47 (18.9%)	202 (81.1%)	
**Stage**						
Stage II	51 (18.7%)	222 (81.3%)	0.239	83 (27.7%)	217 (72.3%)	<0.001
Stage III	23 (14.2%)	139 (85.8%)		22 (12.1%)	160 (87.9%)	
**CDX2^c^**						
Positive	40 (11.6%)	305 (88.4%)	<0.001	66 (17.1%)	321 (82.9%)	<0.001
Negative	21 (61.8%)	13 (38.2%)		27 (69.2%)	12 (30.8%)	
**Location^d^**						
Right side	58 (22.7%)	198 (77.3%)	<0.001	96 (33.9%)	187 (66.1%)	<0.001
Left side	16 (8.9%)	163 (91.1%)		9 (4.5%)	190 (95.5%)	
**Histology**						
Adenoc. NOS	67 (17.0%)	326 (83.0%)	1.000	82 (19.2%)	345 (80.8%)	<0.001
Other^e^	7 (16.7%)	35 (83.3%)		23 (41.8%)	32 (58.2%)	
**Age in years**						
Mean (SD)	68.3 (12.6)	66.8 (12.3)	0.365	68.5 (13.5)	67.3 (12.0)	0.414
**MMR**						
dMMR	40 (45.5%)	48 (54.5%)	<0.001	–	–	–
pMMR	32 (9.7%)	299 (90.3%)		–	–	
**PD-L1**						
Neg (<1%)	–	–	–	48 (13.8%)	299 (86.2%)	<0.001
Pos (≥1%)	–	–		40 (55.6%)	32 (44.4%)	
**CD3+ TILs (intraep.)**						
Low (0 or 1)	10 (5.1%)	185 (94.9%)	<0.001	23 (11.0%)	187 (89.0%)	<0.001
High (2 or 3)	61 (27.6%)	160 (72.4%)		75 (30.9%)	168 (69.1%)	
**CD8+ TILs (intraep.)**						
Low (0 or 1)	11 (5.4%)	192 (94.6%)	<0.001	23 (10.4%)	199 (89.6%)	<0.001
High (2 or 3)	59 (30.9%)	132 (69.1%)		70 (32.7%)	144 (67.3%)	
**CD3+ TILs (stroma)**						
Median score (25/75 percentiles)	20.0 (10.0/30.0)	40.0 (26.3/50.0)	<0.001	32.5 (15.0/45.0)	20.0 (10.0/30.0)	<0.001
**CD8+ TILs (stroma)**						
Median score (25/75 percentiles)	3.0 (1.0/8.0)	10.0 (5.0/24.0)	<0.001	10.0 (3.0/20.0)	5.0 (1.0/10.0)	<0.001

A: PD-L1 expression dichotomized into negative or positive (expression in <1% versus ≥1% of tumor cells) B: For categorical variables: Fisher’s exact test (two sided), for continuous variables: T-test (age), Mann–Whitney U (stromal TILs). C: Positive if CDX2 is expressed in ≥50% of tumor cells. D: Right: Ascending and transverse colon. Left: Descending and sigmoid colon. E: Signet ring cell carcinoma and mucinous adenocarcinoma.

### 3.3 Effect of tumor-infiltrating lymphocytes

**Figure 3:**
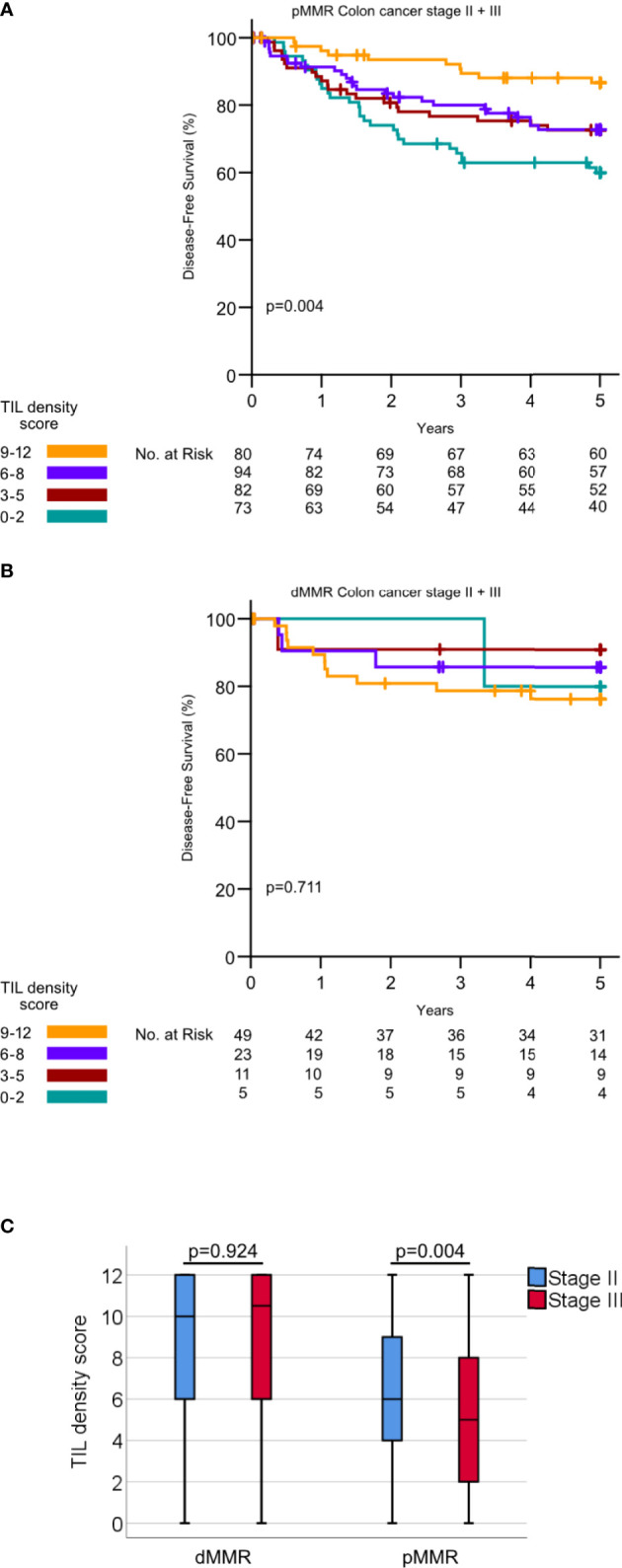
**(A)** Kaplan–Meier curves for DFS for different TIL density scores in pMMR tumors. P-values calculated by log rank test. **(B)** Kaplan–Meier curves for DFS for different TIL density scores in dMMR tumors. P-values calculated by the log-rank test **(C)** TIL density scores in dMMR tumor stage II versus III and pMMR tumors stage II versus III. P-values calculated by Mann–Whitney U-test.

High intraepithelial CD3+ and CD8+ scores and high stromal CD3+ and CD8 + TIL scores were all individually significantly associated with a lower stage, CDX2 negativity, right-sided cancer, the dMMR phenotype, and PD-L1 positivity (**Table 2** and **Supplementary Table 1**). In addition, there was a slightly higher median infiltration of stromal CD3+ TILs in patients aged 67 years or older compared to younger patients (median density 25.0 vs. 20.0, p =0.005, **Supplementary Table 1**). The association between TIL density and age was not observed for CD3+ intraepithelial TILs or CD8+ TILs. TILs scores were all strongly associated with each other (p <0.001 for all comparisons, **Supplementary Table 1**). For pMMR tumors, the total TIL density score was significantly lower in stage III, compared to stage II (**Figure 3C**). In contrast, dMMR tumors had a high total TIL score for both stage II and III. The total TIL score was associated with survival for pMMR tumors but not for dMMR tumors (**Figures 3A, B**). These data suggest that the prognostic difference between dMMR stage II vs. stage III tumors is not explained by a difference in the density of TILs.

### 3.4 Effect of programmed cell death ligand 1 expression

PD-L1 expression in ≥1% of tumor cells was associated with CDX2 negativity, right-sided cancer, dMMR, and a high infiltration of CD3+ and CD8+ intraepithelial and stromal TILs (**Table 2**). In Kaplan–Meier analysis with the log-rank test, PD-L1 expression did not significantly affect survival in stage II and III colon cancer analyzed together (PD-L1-positive: mean DFS 51.7 months (95% CI 47.4-55.9) vs. PD-L1-negative mean DFS 48.6 months (46.5-50.7), p=0.120) (**Supplementary Figure**) or in subgroup analyses.

### 3.5 Prognostic shift of deficient mismatch repair cancers in multivariate Cox regression model

Cox regression analysis models were made for both DFS and OS. The univariate Cox regression model for all variables assessed separately is found in the supplementary material (**Supplementary Table 4**). To determine whether the prognostic effect of MMR depends on the stage, our multivariate Cox regression models included a stage*MMR interaction term. In addition, they were run as split models layered by stage to assess the effect of MMR in stage II and stage III separately (**Table 3**). There were statistically significant interactions between the stage and the MMR phenotype [p<0.001 (DFS), p=0.010 (OS)], indicating that the prognostic impact of the MMR phenotype depends on the stage. The split models both show that in stage III patients, dMMR is a poor prognostic marker [DFS: HR 4.17 (95% CI 2.02-8.61), p<0.001, OS: HR 2.94 (95% CI 1.41-6.13), p=0.004]. In contrast, in stage II patients, there is a tendency toward an improved prognosis for patients with dMMR for DFS (HR 0.24 (95% CI 0.06-1.04) p=0.057) but not for OS (HR 0.80 (95% CI 0.37-1.73), p=0.571). As the indications for administering adjuvant chemotherapy and surgical methods changed between the time of including patients in the two different studies, we adjusted for these differences by adding the variable “cohort” in our model. Decreasing the density of TILs and low CDX2 expression were negative prognostic markers for DFS but not for OS. PD-L1 expression was not associated with prognosis. Our multivariate Cox regression models support that the prognostic impact of dMMR differs between stage II and III.

**Table:** Table 3 Split multivariate Cox regression analysis for disease-free survival and overall survival.

	Disease-Free Survival (DFS)		Overall Survival (OS)	
	Hazard ratio (95% CI)	p-value	Hazard ratio (95% CI)	p-value
Tumor-infiltrating lymphocyte density score (increasing)	0.91 (0.85 - 0.97)	0.008	0.96 (0.90 - 1.03)	0.230
PD-L1 (<1% vs. >1%)	0.75 (0.31 - 1.79)	0.509	1.38 (0.70 - 2.71)	0.355
TNM stage (III vs. II)	3.16 (1.88 - 5.30)	<0.001	2.53 (1.56 - 4.10)	<0.001
CDX2 expression (low vs. high)	2.63 (1.20 - 5.75)	0.015	1.50 (0.70 - 3.17)	0.295
Cohort (HDS vs. NGICG)	0.56 (0.34 - 0.90)	0.017	0.91 (0.60 - 1.39)	0.659
Treatment (surgery only vs. adjuvant chemotherapy)	1.53 (0.95 - 2.47)	0.083	2.27 (1.39 - 3.70)	0.001
Interaction MMR phenotype by stage		<0.001		0.010
dMMR vs. pMMR				
In stage II colon cancer	0.24 (0.06 - 1.04)	0.057	0.80 (0.37 - 1.73)	0.571
In stage III colon cancer	4.17 (2.02 - 8.61)	<0.001	2.94 (1.41 - 6.13)	0.004

## 4 Discussion

Our study demonstrates that the prognostic effect of dMMR differs between colon cancer stage II and stage III. In this study, dMMR was a poor prognostic marker in colon cancer stage III, both for DFS and OS. In stage II, dMMR was a marker of improved prognosis for DFS but not for OS. This stage-dependent difference remains significant when adjusting for the influence of TIL density, PD-L1 expression, CDX2 expression, and administration of adjuvant chemotherapy.

The favorable prognosis associated with dMMR in stage II colon cancer is supported by several studies (15). According to current treatment guidelines, dMMR stage II colon cancer represents a low-risk group of patients who do not need adjuvant chemotherapy. Still, high-risk T4 stage II patients should be considered for adjuvant chemotherapy regardless of the MMR status because of their increased risk of relapse (5). In our study, dMMR in stage II was a marker of improved DFS but not OS. Stage II colon cancer patients have a good prognosis, and many patients are diagnosed at an older age. We therefore believe that DFS is a better reflection of the biology of dMMR stage II tumors than OS. Decreasing percentages of dMMR colon tumors are seen with advancing tumor stage (16). Still, there is increasing evidence that dMMR cancers that do progress to stage IV represent tumors with a more aggressive biology compared to pMMR cancers. For stage IV colorectal cancer, several studies including the consensus molecular subtypes (CMS) classification report that dMMR is a poor prognostic marker (3, 17, 18). Assessing the prognosis of dMMR stage III patients might help us understand at what point in the tumorigenesis that dMMR goes from a favorable to an unfavorable biomarker.

However, results from studies assessing the prognosis of stage III dMMR colon cancers are discrepant. Some report that the dMMR status conveys an improved prognosis in stage II and III analyzed together (19–22), and some report dMMR as a marker of improved prognosis in stage III specifically (1, 20, 21, 23). Other studies report that the prognostic impact of dMMR in localized colon cancer differs between stage II and stage III. In a retrospective single-center cohort consisting of 1,250 patients, Mohan et al. demonstrated a worse DFS for stage III patients with dMMR compared to stage III pMMR patients. In stage I and II, dMMR patients had an improved DFS. Their results indicate a prognostic switch in dMMR colon cancer in line with our findings (24). In other studies, there is a favorable prognostic impact of the dMMR phenotype in stage II but the prognostic effect of dMMR is less prominent or lost in stage III dMMR colon cancer. In a single-center consecutive population-based cohort, the dMMR status conveyed an improved outcome in stage II colorectal cancer but did not impact survival in stage III (25). Klingbiel et al. studied 1,254 patients from the PETACC trial. They found that the positive prognostic effect of dMMR was stronger for stage II patients than for stage III patients. In addition, they reported a statistically significant interaction between the stage and the MMR status (16). Cohen et al. proposed that the impact of dMMR on the prognosis of stage III patients depends on the extent of the lymph node metastasis. In the report by Cohen, dMMR was a positive prognostic factor in stage II patients. In stage III, dMMR was positive prognostic factor in N1 disease but not in N2. In fact, the N2 dMMR group had a worse survival than the N2 pMMR group the first two years after adjuvant treatment but similar long-term survival (26).

The improved prognosis of dMMR patients in stage II has been largely explained by a beneficial immune response. The density of tumor-infiltrating CD3+ and CD8+ lymphocytes has been recognized as a prognostic marker in colon cancer (5) and highly correlated with dMMR (27). In our study, TIL density was a strong prognostic marker in the pMMR subgroup. There was no significant difference in the density of TILs between stage II and III dMMR tumors. Other studies report that TIL density impacts the prognosis of both pMMR and dMMR CRC (28–30). Having a limited number of dMMR tumors with a low density of TILs, our series might be too small to assess the prognostic effect of TIL density in dMMR tumors separately.

The prognostic effect of PD-L1-expression in colon cancer is not fully established (31). Rosenbaum et al. demonstrated reduced cancer specific survival in dMMR patients with positive PD-L1 expression (9). Other studies report a better prognosis for patients with positive PD-L1 expression, especially in the pMMR subgroup (32). We were not able to demonstrate a prognostic impact of PD-L1 expression in any subgroups.

Limitations to this study mainly include the use of TMAs and the lack of a validation cohort. The use of TMA can produce inadequate results if the utilized marker is heterogeneously expressed. The density of TILs is considered a robust marker (8). Although the immunoscore was originally developed for assessment in whole tissue sections, strong correlations between the density of TILs observed in TMAs versus whole tissue sections has been reported in several studies, supporting the use of TMAs to evaluate TIL densities (7, 33, 34). PD-L1 can be heterogeneously expressed, and it was assessed in TMAs only in our study. Still, by setting a low threshold for PD-L1-positive cases (1% positive tumor cells), we believe that we have detected the cases with clinically relevant PD-L1-expression. In this study, the MMR phenotype was assessed by immunohistochemistry, using an MMR panel with four proteins to increase sensitivity (35). This method has a high sensitivity for the detection of dMMR and high concordance with results from PCR-based MSI testing (36). Although MMR proteins are considered to be homogenously expressed with either complete positive or complete negative staining of tumor cells, heterogenous immunohistochemical staining patterns have been reported (37). We therefore validated our MMR protein immunohistochemistry in whole tissue sections in our previous study (10).

The number of published articles specifically evaluating the prognosis of stage III dMMR colon cancer is low. Therefore, it is our opinion that this study adds important information about prognostication in this group of patients. In previous literature on this subject, the prognosis of localized colon cancer is often assessed in stage II and III as one group. However, the present study shows that the prognostic impact of dMMR depends on the stage. Our study would benefit from an external validation, and we hope that future studies will further assess this issue. Having a limited number of patients did not allow for subgroup analysis to assess how the prognostic effect of dMMR affects stage III patients with N1 disease versus patients with N2 disease as discussed in the article by Cohen et al. As there was no significant difference between the number of lymph nodes examined or the number of positive lymph nodes between patients with dMMR and patients with pMMR in our cohort, we do not think that the difference in prognosis between dMMR and pMMR in stage III can be explained by a difference in the severity of lymph node metastasis. We have not performed investigations allowing for separating Lynch syndrome from sporadic dMMR. The cohorts in this study represent different time periods. The patients have received different adjuvant chemotherapy regimens and may have been subjected to different surgical regimes, as reflected in the low number of examined lymph nodes in the NGICG cohort. The distribution of other clinicopathological variables is similar between the two cohorts. We have adjusted statistically for the difference between cohorts by including the variable “cohort” in our multivariate Cox regression models.

The reason for the prognostic shift of dMMR colon cancer during tumor progression needs to be studied further. Tumorigenesis, including dMMR colon cancer, are driven by both genetic and epigenetic changes in the colonic epithelium and interactions with the tumor microenvironment. dMMR colon tumors are hypermutated and associated with features that, in isolation, make a poor prognosis. We propose that the prognosis of dMMR tumors depends on the balance between the influence of favorable tumor–host interactions and unfavorable genetic and epigenetic alterations. When the favorable interaction between tumor and the microenvironment is revoked or decreased, the unfavorable influence of poor prognostic markers associated with dMMR tumors will dominate the diagnosis.

This is illustrated in our recently published study, where we describe the interplay between the MMR status, tumor grade, and expression of cell maturation marker CDX2 (caudal type homeobox 2). The loss of CDX2 expression and low tumor grade in isolation are regarded as poor prognostic features. We show that there is a large overlap between dMMR and the loss of CDX2 expression and between dMMR and a high tumor grade. Still, the poor prognostic effect of these markers is restricted to the pMMR group (10). Our current study advances our previous findings by assessing the prognosis of dMMR stage II versus stage III while adjusting for the prognostic impact of the most established immune markers. To our knowledge, this is the first study to show that the prognostic impact of dMMR differs between stage II and III colon cancer also when adjusting for the impact of TIL density and PD-L1 expression.

Treatment with anti-PD-1/PD-L1 antibodies yield large tumor responses in metastatic dMMR colon cancer (38). More recently, the striking effects of neoadjuvant immunotherapy have been demonstrated for early-stage dMMR colon cancer (39–41). There are several ongoing studies on the effects of adjuvant (chemo)immunotherapy in early-stage colon cancer (42). As treatment with immunotherapy is expensive and associated with side effects (43), the expected prognostic outcome in dMMR colon cancer needs to be established before treatment guidelines can be proposed. We believe that the clinical picture for dMMR stage III tumors is far more complex than for dMMR stage II. More comprehensive prospective studies are needed to corroborate the results in our study. Still, this study supports further analyses of the effect of adjuvant immunotherapy in this group of patients.

This article describes the prognostic shift in dMMR colon cancer. We conclude that despite being a marker of improved prognosis in stage II, dMMR is not associated with a favorable prognosis in stage III colon cancer.

## Data availability statement

Data will be made available by reasonable request. Requests to access the datasets should be directed to Olav.dahl@helse-bergen.no.

## Ethics statement

The studies involving human participants were reviewed and approved by The Regional Committee for Medical Research Ethics of Western Norway. All patients have signed written, informed consents for study participation.

## Author contributions

KEH: conceptualization, writing – original draft and editing, CD3 IHC staining, MMR-score, statistical analyses. NBR: CD3 IHC staining, MMR-score, manuscript revision LS: database editing, manuscript revision. OD: project administration, funding acquisition, acquiring of patient materials, manuscript editing. MPM: project management, manuscript editing, conceptualization. All authors contributed to the article and approved the submitted version.

## Funding

For this study, the authors received funding from Health Region West, Norway, University of Bergen, Norway, and the Norwegian Cancer Society.

## Acknowledgments

We thank the Mohn Laboratory for Cancer Research for outstanding facilities and the Department of Pathology Ålesund for MMR protein staining. We want to thank Ole Johnny Steffensen and Yvonne Müller for excellent technical support.

## Conflict of interest

The authors declare that the research was conducted in the absence of any commercial or financial relationships that could be construed as a potential conflict of interest.

## Publisher’s note

All claims expressed in this article are solely those of the authors and do not necessarily represent those of their affiliated organizations, or those of the publisher, the editors and the reviewers. Any product that may be evaluated in this article, or claim that may be made by its manufacturer, is not guaranteed or endorsed by the publisher.
