# Supplementary material for: Stage-dependent prognostic shift in mismatch repair-deficient tumors: Assessing patient outcomes in stage II and III colon cancer
Source: Front Oncol. 2022 Aug 30;12:853545. doi: 10.3389/fonc.2022.853545 (PMC9468812; doi:10.3389/fonc.2022.853545)
Supplement: Supplementary file 1 [file DataSheet_1.pdf]

VARIABLE	CD3+ TILs (intraepithelial) n=470			CD8+ TILs (intraepithelial) n=452			CD3+ TILs (stroma)		CD8+ TILs (stroma)	
	Low (0 or 1) n (%)	High (2 or 3) n (%)	p-value[a]	Low (0 or 1) n (%)	High (2 or 3) n (%)	p-value[a]	Median (25/75 percentiles)	p-value[b]	Median (25/75 percentiles)	p-value[b]
**Sex**										
Female	109 (47.0%)	123 (53.0%)	0.711	115 (51.3%)	109 (48.7%)	0.708	25.0 (10.0/40.0)	0.335	5.0 (2.0/10.0)	0.438
Male	107 (45.0%)	131 (55.0%)		113 (49.6%)	115 (50.4%)		25.0 (10.0/35.0)		5.0 (1.0/10.0)	
**Stage**										
Stage II	122 (40.7%)	178 (59.3%)	0.002	131 (45.6%)	156 (54.4%)	0.008	25.0 (10.0/40.0)	0.001	5.0 (2.0/10.0)	0.002
Stage III	94 (55.3%)	76 (44.7%)		97 (58.8%)	68 (41.2%)		20.0 (8.5/30.0)		3.0 (2.0/7.3)	
**CDX2**[c]										
Positive	188 (50.3%)	186 (49.7%)	0.001	196 (53.8%)	168 (46.2%)	0.018	20.0 (10.0/35.0)	0.002	5.0 (2.0/10.0)	0.014
Negative	8 (21.1%)	30 (78.9%)		13 (33.3%)	26 (66.7%)		35.0 (17.5/45.0)		10.0 (2.0/20.0)	
**Location**[d]										
Right side	114 (41.6%)	160 (58.4%)	0.031	118 (44.2%)	149 (55.8%)	0.002	25.0 (10.0/40.0)	0.001	5.0 (2.0/10.0)	<0.001
Left side	102 (50.2%)	94 (48.0%)		110 (59.5%)	75 (40.5%)		20.0 (10.0/30.0)		3.0 (1.0/9.0)	
**Histology**										
Adenoc. NOS	197 (46.5%)	227 (53.5%)	0.537	202 (49.8%)	204 (50.2%)	0.438	25.0 (10.0/35.0)	0.679	5.0 (2.0/10.0)	0.974
Other[e]	19 (41.3%)	27 (24.9%)		26 (56.5%)	20 (43.5%)		20.0 (10.0/35.0)		3.0 (2.0/10.0)	
**Age in years**										
Mean (SD)	68.2 (12.6)	66.9 (12.1)	0.288	67.8 (11.8)	67.3 (13.1)	0.658				
<67 years							20.0 (10.0/35.0)	0.005	5.0 (2.0/10.0)	0.272
≥67 years							25.0 (10.0/40.0)		5.0 (2.0/10.0)	
**CD3+ TILs IntEp**[f]										
Low (0 or 1)	-	-		163 (81.1%)	38 (18.9%)	<0.001	10.0 (5.0/25.0)	<0.001	2.0 (1.0/5.0)	<0.001
High (2 or 3)	-	-		57 (24.1%)	180 (75.9%)		30.0 (20.0/40.0)		8.0 (3.0/15.0)	
**CD8+ TILs IntEp**[f]										
Low (0 or 1)	163 (74.1%)	57 (25.9%)	<0.001	-	-		15.0 (5.0/25.0)	<0.001	2.0 (1.0/5.0)	<0.001
High (2 or 3)	38 (17.4%)	180 (82.6%)		-	-		30.0 (20.0/40.0)		10.0 (5.0/15.0)	
**CD3+ TILs Stroma**										
Median (25/75 perc)	10.0 (5/25)	30.0 (20/40)	<0.001	15.0 (5/25	30.0 (20/40)	<0.001	**Stromal CD8 TILs**[g]		**Stromal CD3 TILs**[g]	
**CD8+ TILs Stroma**							Low: 10.0 (5/25), p<0.001[b]		Low: 2.0 (1/5), p<0.001[b]	
Median (25/75 perc)	2.0 (1/5)	8.0 (3/15)	<0.001	2.0 (1/5)	10.0 (5/15)	<0.001	High: 30.0 (20/40)		High: 10.0 (5/15)	

Supplementary table 1: Associations between intraepithelial and stromal CD3+ and CD8+ tumor infiltrating lymphocytes and other markers and each other.

A: P-values: Fisher's Exact Test (2-sided). B: Mann-Whitney U. C: Positive if CDX2 is expressed in ≥ 50% of tumor cells. D: Right: Ascending and transverse colon. Left: Descending and sigmoid colon. E: Signet ring cell carcinoma and mucinous adenocarcinoma. F: Intraepithelial. G: Score dichotomized at median.

Supplementary table 2: Goodness of fit test

VARIABLES	NUMBER OF CASES						
	**Original cohort (n=544)**	**Valid PD-L1-staining (n=435)**		**Valid CD3-staining (n=470)**		**Valid CD8-staining (n=452)**	
	Observed	Observed/ expected	p-value	Observed /expected	p-value	Observed /expected	p-value
**Sex**							
Female	270	206/216	0.338	232/233	0.926	224/224	1.000
Male	274	229/219		238/237		228/228	
**Stage**							
UJCC stage II	338	273/270	0.767	300/292	0.447	287/281	0.561
UJCC stage III	206	162/165		170/178		165/171	
**Chemo.**							
Adjuvant chemo.	189	150/151	0.875	159/163	0.674	156/157	0.894
No chemo.	354	285/283		311/306		296/294	
**Histology**							
Adenoc. NOS	479	393/383	0.138	424/414	0.154	406/398	0.246
Other	65	42/52		46/56		46/54	
**Tumor grade**							
Low grade	430	350/343	0.399	379/372	0.415	365/357	0.343
High grade	107	79/86		85/92		81/89	
**Tumor location**							
Right side	325	256/260	0.680	274/281	0.510	267/270	0.774
Left side	219	179/175		196/189		185/182	
**Cohort**							
NGICG	276	235/220	0.177	236/238	0.854	226/229	0.778
Haraldsplass	268	200/214		234/232		226/223	
**MMR**							
dMMR	105	88/91	0.686	98/99	0.922	93/87	0.832
pMMR	377	331/328		355/354		343/313	
**CDX2**							
CDX2 negativ	41	34/35	0.814	38/37	0.966	39/37	0.761
CDX2 positive	402	345/344		374/374		364/366	
**Age in years** mean (SD)	67.5 (12.32)	67.1 (12.33)	0.630	67.5 (12.36)	0.971	67.51 (12.41)	0.961

Method: Chi square test (Student's t-for Age) with missing case-adjustment for expected values for MMR and CDX2. Goodness of fit test for the MMR staining is found in Hestetun et al. 2020 (13), Supplementary.

Supplementary Table 3: Comparing cohorts

	NGICG-cohort (n=276)	HDH-cohort (n=268)	p-value
**Age, mean/range**	61.7 (28.4-75.1)	73.5 (28.0-93.0)	<0.001
**Sex**			
Female	131 (47.5%)	125 (46.6%)	0.784
Male	145 (52.5%)	143 (53.4%)	
**Stage**			
UJCC stage II	174 (63.0%)	164 (61.2%)	0.528
UJCC stage III	102 (37.0%)	104 (38.8%)	
**Chemotherapy**[a]			
Adjuvant chemo.	140 (50.7%)	49 (18.3%)	<0.001
No chemotherapy	136 (49.3%)	218 (81.3%)	
**Histology**			
Adenocarcinoma NOS	242 (87.7%)	237 (88.4%)	0.696
Mucinous or signet	34 (12.3%)	31 (11.6%)	
**Tumor grade**[b]			
Low tumor grade	220 (79.7%)	210 (78.4%)	0.506
High tumor grade	52 (18.8%)	55 (20.5%)	
**MMR-phenotype**[c]			
dMMR	49 (17.8%)	56 (20.9%)	0.286
pMMR	192 (69.6%)	185 (69.0%)	
**Tumor location**[d]			
Right side	152 (55.1%)	173 (64.6%)	0.001
Left side	124 (44.9%)	95 (35.4%)	

Supplementary table 3: Table comparing the two cohorts using chi square test (T test for Age). a: Data missing from one patient in HDH cohort. b: Data missing from 4 patients in NGICG cohort and 3 patients in HDH cohort. c: Results missing from 35 patients in NGICG cohort and 27 patients in the HDH cohort. d: Right side: Ascending and transverse colon. Left side: Descending and sigmoid colon

Supplementary Figure:

Kaplan Meier curves comparing Disease-Free Survival between PD-L1 negative and PD-L1 positive cases for stage II and stage III colon cancer. p-values calculated by log rank test.

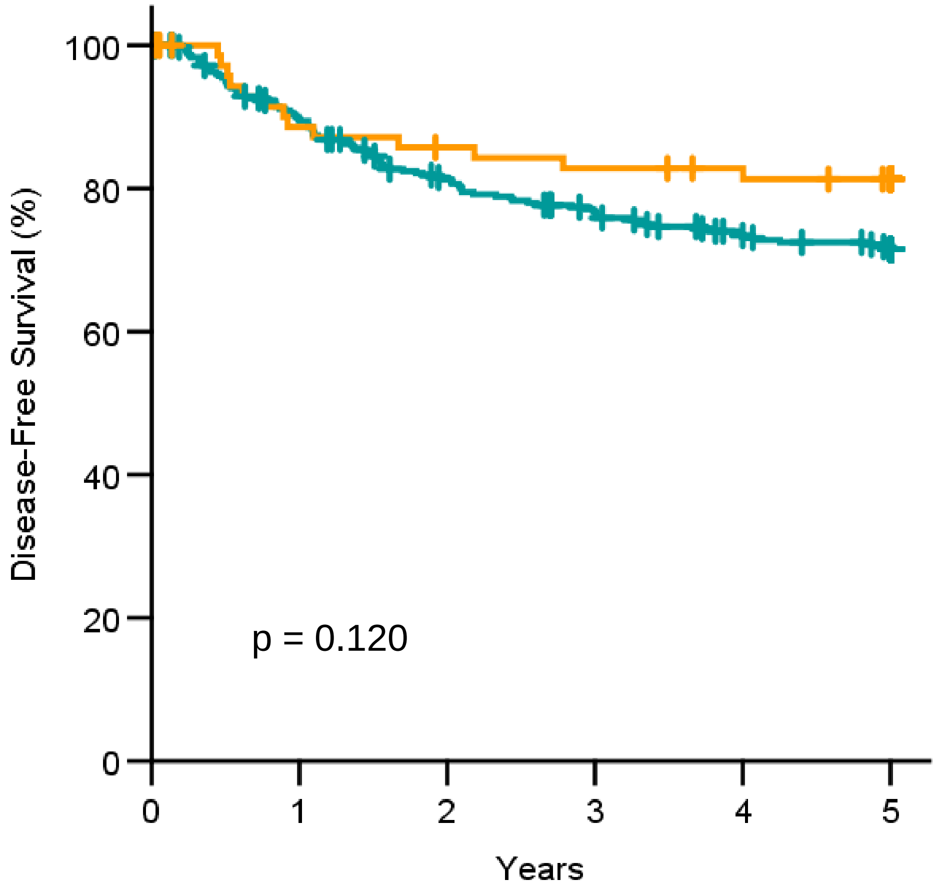

PD-L1 negative

PD-L1 positive

No. at Risk					
359	311	274	254	235	223
74	62	59	57	55	51

REMARK CHECKLIST	COMMENT
**Introduction**	
State the marker examined, the study objectives, and any pre-specified hypotheses.	Described in introduction.
**Patients**	
Describe the characteristics (for example disease stage or co-morbidities) of the study patients, including their source and inclusion and exclusion criteria.	Described in material and methods and table 1.
Describe treatments received and how chosen (for example, randomized or rule-based).	Described in material and methods.
**Specimen characteristics**	
Describe the type of biological material used (including control samples) and methods of preservation and storage	Described in material and methods.
**Assay methods**	
Specify the assay method used and provide (or reference) a detailed protocol including specific reagents or kit used, quality control procedures, reproducibility assessments, quantitation methods, and scoring and reporting protocols. Specify whether and how assays were performed blinded to the study endpoint.	Relevant information on IHC methods are provided in the material and methods section.
**Study design**	
State the method of case selection, including whether prospective or retrospective and whether stratification or matching (for example, by stage or disease or age) was used. Specify the time period from which cases were taken, the end of the follow-up period, and the median follow-up time.	Please refer to original studies by Olav Dahl et. al and Luka Stanisavljević
Precisely define all clinical endpoints examined.	Described in material and methods
List all candidate variables initially examined or considered for inclusion in models.	Relevant clinicopathological variables were included in the models.
Give rationale for sample size; if the study was designed to detect a specified effect size, given the target poser and effect size.	Not applicable.
**Statistical analysis methods**	
Specify all statistical methods, including details of any variable selection procedures and other model-building issues, how model assumptions were verified, and how missing data were handled.	Model building and variable selection is described in material and methods. Goodness of fit-analyses are available in supplementary.
Clarify how marker values were handled in the analysis; if relevant, describe methods used for cutpoint determination	Described in the material and methods section.

**Data**	
Describe the flow of patients through the study, including number of patients included in each stage of the analysis (a diagram may be helpful) and reasons for dropout. Specifically. Both overall and for each subgroup extensively examined report the number of patients and number of events.	Please refer to original studies by Olav Dahl et. al and Luka Stanisavljević
Report distributions of basic demographic characteristics (at least age and sex), standard (disease-specific) prognostic variables and tumor marker, including numbers of missing values.	Provided in table 1.
**Analysis and presentation**	
Show the relation of the marker to standard prognostic variables.	Provided in table 2.
Present univariable analyses showing the relation between the marker and outcome, with the estimated effect (for example hazard ration and survival probability). Preferably provide similar analyses for all other variables being analyzed. For effect of a tumor marker on a time-to-event outcome, a Kaplan-Meier plot is recommended.	Kaplan Meier plots are provided in figures 2 and 3.
For key multivariable analyses, report estimated effects (for example, hazard ratio) with confidence intervals for the marker and, at least for the final model, all other variables in the model.	Provided in table 3.
Among reported results, provide estimated effects with confidence intervals from an analysis in which the marker and standard prognostic variables are included, regardless of their statistical significance.	Provided in table 3.
If done, report results of further investigations, such as checking assumptions, sensitivity analyses, and internal validation.	Assumptions for cox model were checked as described in the material and methods section and internal validation of MMR-IHC is described in previously published article (reference number 10).
**Discussion**	
Interpret the results in the context of the pre-specified hypotheses and other relevant studies; include a discussion of the limitations of the study.	Provided in discussion section.
Discuss implications for future research and clinical value.	Described in discussion and conclusion.

	Disease-free survival (DFS)		Overall Survival (OS)	
	**Hazard ratio (95% CI)**	**p value**	**Hazard ratio (95% CI)**	**p value**
Tumor infiltrating lymphocyte density score (increasing)	0.91 (0.86-0.96)	<0.001	0.96 (0.92-1.01)	*0.148*
PD-L1 (<1% vs. >1%)	1.58 (0.88-2.81)	0.124	0.97 (0.61-1.56)	0.910
TNM stage (III vs. II)	4.04 (2.85-5.74)	<0.001	2.91 (2.11-4.03)	<0.001
CDX2 expression (low vs. high)	1.64 (0.96-2.84)	0.072	1.65 (0.99-2.75)	0.055
Cohort (HDS vs. NGICG)	0.78 (0.56-1.10)	0.165	1.08 (0.79-1.49)	0.631
Treatment (Surgery only vs. adjuvant chemotherapy)	0.84 (0.60-1.19)	0.328	0.79 (0.56-1.12)	0.187
MMR (pMMR vs. dMMR)	1.56 (0.95-2.53)	0.077	1.01 (0.68-1.52)	0.957

Supplementary Table 4: **Univariate** Cox regression analysis (for each variable included in table 3 separately).
